# Role of circulating free DNA in evaluating clinical tumor burden and predicting survival in Chinese metastatic colorectal cancer patients

**DOI:** 10.1186/s12885-020-07516-7

**Published:** 2020-10-16

**Authors:** Xiaojing Xu, Yiyi Yu, Minna Shen, Mengling Liu, Shengchao Wu, Li Liang, Fei Huang, Chenlu Zhang, Wei Guo, Tianshu Liu

**Affiliations:** 1 Department of Oncology, Zhongshan Hospital, Fudan University, 180 Feng Lin Road, Shanghai, 200032 PR China; 2 Department of Laboratory Medicine, Zhongshan Hospital, Fudan University, 180 Feng Lin Road, Shanghai, 200032 PR China

**Keywords:** Metastatic colorectal Cancer (mCRC), Circulating free DNA, Tumor burden, Survival, Metastasis

## Abstract

**Background:**

The aim of this study was to explore the utility of circulating free DNA (cfDNA) in the evaluation of clinical tumor burden and survival in Chinese patients with metastatic colorectal cancer (mCRC) and to preliminarily summarize some metastatic characteristics associated with mutational status.

**Methods:**

A panel covering a total of 197 hotspot mutations of *KRAS, NRAS, BRAF* and *PIK3CA* was used to evaluate the mutational status in plasma by next-generation sequencing (NGS) technology in 126 patients with mCRC. An amplification-refractory mutation system (ARMS) was used to analyze genomic DNA from matched tissue samples. Clinical markers including carcinoembryonic antigen (CEA), carbohydrate antigen 199 (CA199), carbohydrate antigen 125 (CA125), neuron-specific enolase (NSE) and lactate dehydrogenase (LDH) in serum and the sum of all tumor diameters on CT or PET/CT were collected to indicate clinical tumor burden. The correlations between cfDNA and clinical tumor burden were analyzed using Pearson correlation and linear regression models. The median progression-free survival (PFS) and 1-year overall survival (OS) rates were calculated by Kaplan-Meier (K-M) survival analysis.

**Results:**

Of the 126 enrolled patients, patients who were tested positive for mutations in plasma accounted for 45.2% (57/126). Mutations in *KRAS, NRAS, BRAF* and *PIK3CA* were detected in 37.3% (47/126), 1.6% (2/126), 3.2% (4/126) and 13.5% (17/126) of patients, respectively. The overall concordance rate of mutational status between plasma and matched tissues was 78.6% (99/126). Sixteen patients had mutations in plasma that were not detected in tissue, including some rare hotspot mutations. The cfDNA concentration was significantly correlated with the levels of clinical markers, especially CEA (*P* < 0.0001, Pearson *r* = 0.81), LDH (*P* < 0.0001, Pearson *r* = 0.84) and the sum of tumor diameters (*P* < 0.0001, Pearson *r* = 0.80). Patients with a high cfDNA concentration (> 17.91 ng/ml) had shorter median progression-free survival (6.6 versus 11.7 months, *P* < 0.0001) and lower 1-year overall survival rate (56% versus 94%, *P* < 0.0001) than those with a low cfDNA concentration (≤17.91 ng/ml). The most common metastatic site was the liver (77.8%), followed by the lymph nodes (62.7%), lung (40.5%), peritoneum (14.3%) and bone (10.3%), in all patients. There was no significant difference in metastasis between different mutational statuses.

**Conclusion:**

Analyzing mutations in plasma could provide a more comprehensive overview of the mutational landscape than analyzing mutations in tissue. The cfDNA concentration could be a quantitative biomarker of tumor burden and could predict survival in Chinese patients with mCRC.

## Background

Colorectal cancer (CRC) is the fourth leading cause of cancer-related death worldwide, and the incidence has increased in the past decade with an aging and growing population [1]. With the development of liquid biopsy strategies, circulating free DNA (cfDNA) derived from plasma, consisting of healthy and tumor DNA (circulating tumor DNA, ctDNA), has been increasingly investigated as a promising biomarker for cancer management [2]. Sequencing of plasma samples may improve diagnostic efficiency and preemptively predict recurrence and treatment response in CRC [3]. Furthermore, monitoring genomic alterations in *RAS*, *BRAF* and other cancer-related genes in plasma can guide targeted therapeutic strategies and especially optimize anti-epidermal growth factor receptor (EGFR) therapy for metastatic CRC (mCRC) [4, 5].

The change in tumor burden is an essential feature in the clinical evaluation of cancer therapy. However, there is no clear definition of or uniform assessment method for determining overall tumor burden. The imaging-based Response Evaluation Criteria in Solid Tumors (RECIST) are widely used to measure partial disease load and to evaluate sequential treatment responses based on the assessment of anatomical tumor burden [6]. In addition, circulating biomarkers and clinical symptoms are also significant for the effective surveillance of tumor burden in clinical practice. Such serum biomarkers include carcinoembryonic antigen (CEA), carbohydrate antigen 199 (CA199), carbohydrate antigen 125 (CA125), neuron-specific enolase (NSE), lactate dehydrogenase (LDH), etc. The potential of exploiting quantified levels and mutational load of cfDNA to monitor tumor burden in CRC patients has also been reported in a few studies [7, 8].

The aims of this study were to further explore the mutational signature in plasma and the utility of cfDNA in the evaluation of tumor burden and survival in Chinese patients with mCRC. We also summarized and preliminarily analyzed some metastatic characteristics according to mutations in plasma.

## Methods

### Patients

Patients with mCRC treated in the Oncology Department, ZhongShan Hospital, Fudan University, Shanghai, China, from April to November 2018 were enrolled in this study. The inclusion criteria were adenocarcinoma in the colon or rectum, recurrent or primary metastatic disease, planned treatment with first-line chemotherapy, life expectancy more than 3 months, age > 18 years, and Eastern Cooperative Oncology Group performance status 0–1. Plasma samples were collected at baseline. This study was approved by the ethics committee of Zhongshan Hospital. Written informed consent was obtained from all patients.

### Sample collection, DNA extraction and sequencing of plasma samples

A total of 20 ml of venous blood was collected from each patient and then centrifuged at 1900×*g* for 10 min at 4 °C to separate the plasma from peripheral blood cells within 2 h of receipt. Plasma was then further centrifuged at 16,000×*g* for 10 min at 4 °C to pellet any remaining cells. The cfDNA was extracted from 8.0 mL of plasma using the QIAamp Circulating Nucleic Acid Kit (Qiagen, 55,114) according to the manufacturer’s instructions and quantified using the Qubit Fluorometer 3.0 (Life Technologies, Grand Island, NY) and 2100 bioanalyzer (Technologies, Palo Alto, CA). The quality control criteria for cfDNA were as follows: the concentration of the cfDNA was less than 0.9 ng/μl as measured by the Qubit dsDNA HS Assay Kit (Thermo Fisher Scientific, Q32854); the total amount of cfDNA was greater than 20 ng; the fragment distribution feature detected by the 2100 bioanalyzer had a typical peak at 160–180 bp and a small peak at 320–360 bp. The extracted cfDNA was immediately subjected to the next step or cryopreservation. The cfDNA was stored at − 20 °C for no more than 1 week; for long-term storage, the DNA was placed at − 80 °C for no more than 6 months. Attempts were made to keep the number of repeated freeze-thaw cycles of cfDNA samples ≤3.

Plasma cfDNA (20 ng) was subjected to Firefly™ amplicon-based next-generation sequencing (NGS) technology using Accu-Kit™ CRC-01 (AccuraGen, Shanghai, China), as described in a previous study [9]. The panel covered a total of 197 hotspot mutations in exons 2, 3 and 4 of *KRAS*; exons 2, 3 and 4 of *NRAS*; exons 9 and 20 of *PIK3CA*; and exon 15 of *BRAF* (Supplemental Table 1). NGS libraries were generated using the KAPA Sequencing Library Construction Kit (Kapa Biosystems, Boston, MA, USA). The quality control criteria for the cfDNA libraries were as follows: the concentration of the library was less than 0.5 ng/μl as measured by the Qubit dsDNA HS Assay Kit (Thermo Fisher Scientific, Q32854); the fragment distribution feature detected by the 2100 bioanalyzer had a typical peak at 600–1300 bp, and the average length was 900–1000 bp. Libraries were then sequenced using 2 × 250 pair-end reads on an Illumina MiSeq Dx sequencer (Illumina, San Diego, CA, USA). Unique sequencing reads were determined by using an AccuraGen proprietary algorithm including quality control, read collapse, read alignment, and variant calling. The average coverage depth for all probes in plasma was nearly 30,000×, and the sensitivity of variant detection in this study was 0.2%. For details of this laboratory-developed test, refer to the reported articles [10]. Generally, sequence reads were aligned to the hg19/GRCh37 human reference sequence, and background noise introduced by random NGS error was removed by AccuraGen proprietary algorithms. The firefly algorithm used a 2-proportion Z-test [11] to reduce false positive mutations from background noise, and mutations passing the Z-test were reported. The cfDNA concentration was determined by the Qubit Fluorometer 3.0.

### Identification of mutations in tissue samples

The isolation and purification of genomic DNA from formalin-fixed, paraffin-embedded (FFPE) tissue sections was performed using an AmoyDx® FFPE DNA Kit (Xiamen, China). FFPE specimen tissue sections were first deparaffinized with the xylene/ethanol method and then incubated in buffer DTL and proteinase K solution to release DNA from the sections. A short incubation in DES buffer at a high temperature partially reversed formalin crosslinking of the released nucleic acids, improving DNA yield and quality as well as DNA performance in downstream assays. The lysate was mixed with DTB buffer and ethanol to provide appropriate binding conditions for DNA. Then, the mixture was applied to a DNA spin column, where the DNA bound to the membrane and impurities were removed with wash buffer. The DNA was eluted in DTE buffer. A human *KRAS*/*NRAS*/*BRAF*/*PIK3CA* gene mutation fluorescence polymerase chain reaction (PCR) diagnostic kit (Amoy Diagnostics, Xiamen, China) based on amplification refractory mutation system (ARMS) technology approved by the China Food and Drug Administration was used to analyze genomic DNA from tissue samples. The list of mutations detectable with this panel is shown in Supplemental Table 2. The assay was carried out according to the manufacturer’s protocol for the ABI7500 real-time PCR system (Thermo Fisher Scientific, Waltham, MA, USA). We defined a cut-off of 1% mutation content as a sample quality check according to the minimum requirement of ARMS technology (approximately 1% analytical sensitivity).

### Assessment of serum markers

Carcinoembryonic antigen (CEA) was measured using an enzyme-linked immunosorbent assay (CanAg CEA EIA). Carbohydrate antigen 199 (CA199), carbohydrate antigen 125 (CA125), and neuron-specific enolase (NSE) were measured by electrochemical fluoroimmunoassay (Roche Elecsys series). Lactate dehydrogenase (LDH) was detected by an LDH Assay Kit (Abcam).

### Statistical methods

The correlations between clinical factors and cfDNA levels were analyzed using *Pearson* correlation and linear regression models. The optimal cutoff level for cfDNA was determined by receiver operating curve (ROC) analysis. Median progression-free survival (PFS) and 1-year overall survival (OS) were calculated by Kaplan-Meier (K-M) survival analysis, and the significance was evaluated by the log-rank test. Tumor metastatic sites with different gene statuses were compared using the chi square test. Statistical tests provided two-sided *P* values, and a significance level of *P* < 0.05 was used. Statistical analyses were carried out using SPSS 24.

## Results

### Patient characteristics

**Fig. 1:**
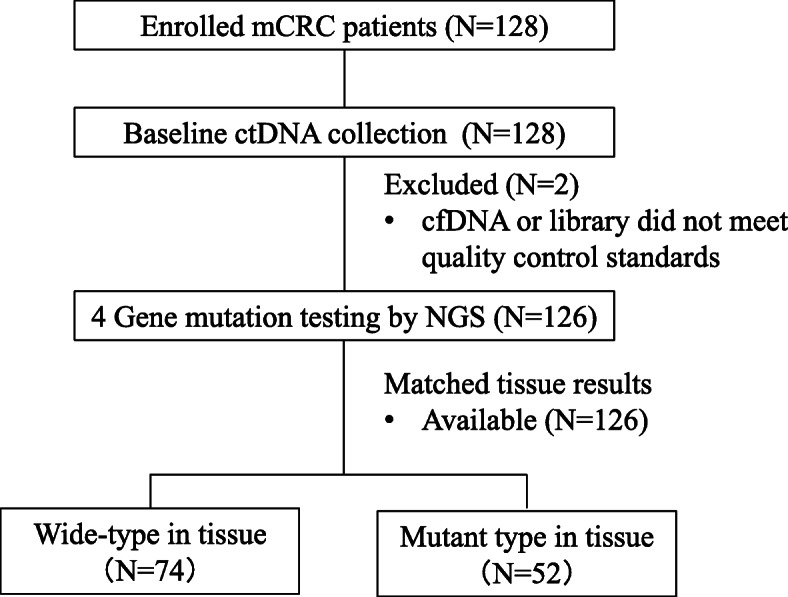
Overview of patient enrollment

A total of 128 patients were enrolled in this study. Because the cfDNA or the library did not meet the quality control standards in 2 patients, blood samples from 126 patients were collected for analysis of four genes *(KRAS, NRAS, BRAF* and *PIK3CA*) before treatment. All patients had gene results from matched tissues. The flow chart is shown in Fig. 1. The clinical characteristics are summarized in Table 1. The median age was 58 years (26–84 years). There were 90 males and 36 females. The most common locations of primary lesions were the sigmoid colon and rectum (38.9 and 35.7%, respectively). Ninety-one (72.2%) patients had received surgery before.

**Table 1:** Baseline Characteristics

Characteristics	No. (%) (***N*** = 126)
Median Age (years,range)	58 (26–84)
Gender	
Male	90 (71.4)
Female	36 (28.6)
Primary site	
Ascending colon	18 (14.3)
Transverse colon	7 (5.6)
Descending colon	7 (5.6)
Sigmoid colon	49 (38.9)
Rectum	45 (35.7)
Surgery	
No surgery	35 (27.8)
Primary tumor resection	48 (38.1)
Primary tumor and liver metastase resection	43 (34.1)
Metastatic site	
Liver	98 (77.8)
Lymph node	79 (62.7)
Lung	51 (40.5)
Peritoneum	18 (14.3)
Bone	13 (10.3)
Others	13 (10.3)
Metastatic organ number	
One	46 (36.5)
Multi	80 (63.5)

### Mutations in plasma and tissues

The results showed that 69 patients (54.8%) had wild-type DNA and 57 patients (45.2%) had gene mutations in plasma (Table 2). Mutations of *KRAS, NRAS, BRAF* and *PIK3CA* were detected in 37.3% (47/126), 1.6% (2/126), 3.2% (4/126) and 13.5% (17/126) of patients, respectively. Polyclonal mutations were detected in 13 patients (10.3%), which were present mostly in the form of double mutations in *KRAS/PIK3CA* or *NRAS/PIK3CA*. The most common mutation sites were G12 (52.6%) and G13 (12.3%) in exon 2 of the *KRAS* gene. Some rare mutations, including the V14I mutation in exon 2 of *KRAS* and the K117N and K117R mutations in exon 4 of *KRAS,* were detected in this study (Table 3 and Fig. 3a). In matched tissue samples, 74 patients (58.7%) had no mutations and 52 patients (41.3%) had mutations, including *KRAS* mutations in 42 patients (33.3%), *NRAS* mutations in 3 patients (2.4%), *BRAF* mutations in 3 patients (2.4%) and *PIK3CA* mutations in 5 patients (4.0%). The concordance rate of mutational status between plasma and tissue was 78.6%. Of the 27 patients with inconsistency, 11 patients had mutations in tissue that were not detected in plasma, and 16 patients had mutations in plasma that were not detected in tissue.

**Table 2:** Mutational status in plasma and tissue

Mutational status	No. (%)	
	Plasma (***N*** = 126)	Tissue (***N*** = 126)
Wild-type	69 (54.8)	74 (58.7)
*KRAS* mutation	35 (27.8)	41 (32.5)
*NRAS* mutation	1 (0.8)	3 (2.4)
*BRAF* mutation	4 (3.2)	3 (2.4)
*PIK3CA* mutation	4 (3.2)	4 (3.2)
*KRAS + PIK3CA* mutations	12 (9.5)	1 (0.9)
*NRAS + PIK3CA* mutations	1 (0.8)	0 (0)

**Table 3:** Mutations detected in plasma

Gene	Exon	Mutational hotspots	No. (%) (*N* = 57)
*KRAS*	2	G12D	10 (17.5)
		G12V	10 (17.5)
		G13D	5 (8.8)
		G12A	4 (7.0)
		G12S	3 (5.3)
		G12C	2 (3.5)
		G13C	1 (1.8)
		G13V	1 (1.8)
		V14I + G12A	1 (1.8)
	3	Q61H	4 (7.0)
	4	A146T	4 (7.0)
		K117N	1 (1.8)
		K117R	1 (1.8)
*NRAS*	3	Q61K	1 (1.8)
*PIK3CA*	9	E545K	5 (8.8)
		E542K	3 (5.3)
		T544I	1 (1.8)
		D549N	1 (1.8)
		Q546K	1 (1.8)
		Q546P	1 (1.8)
		E545G	1 (1.8)
	20	H1047R	2 (3.5)
		H1047Y	1 (1.8)
*BRAF*	15	V600E	3 (5.3)
		K601E	1 (1.8)

### Correlation between cfDNA and tumor burden

**Fig. 2:**
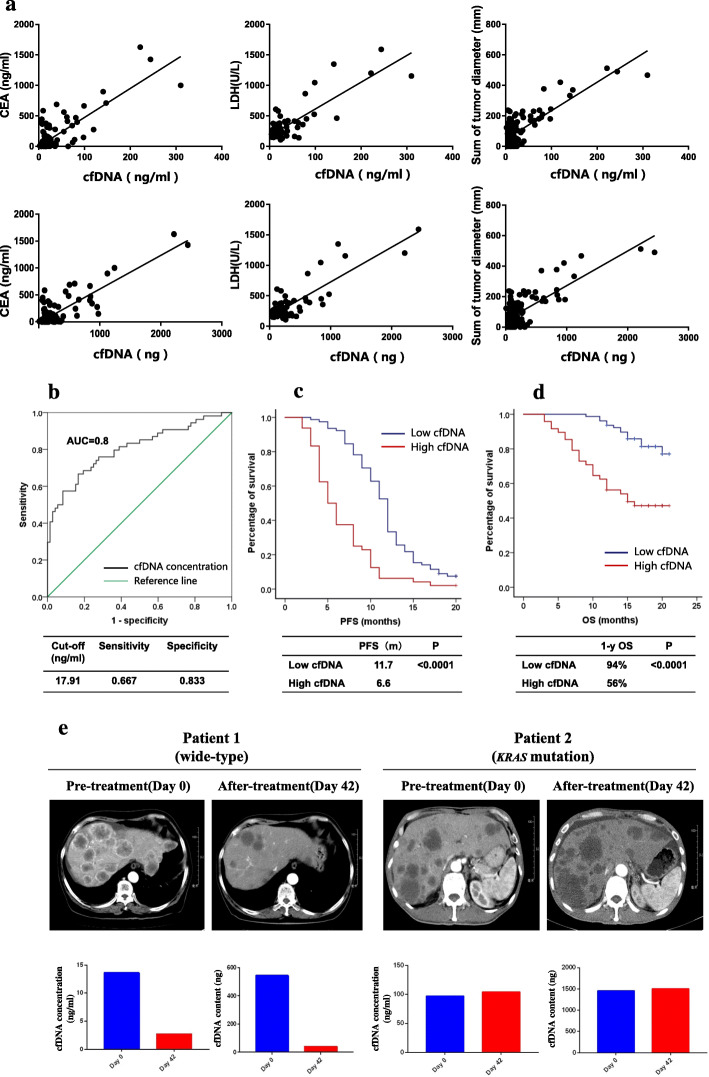
Correlation between cfDNA and tumor burden. **a** The concentration and content of cfDNA were significantly correlated with the levels of CEA and LDH and the sum of tumor diameters. **b** Through ROC curve analysis, the optimal cutoff level for cfDNA concentration was determined to be 17.91 ng/ml. **c** The median progression-free survival (11.7 versus 6.6 months, *P* < 0.0001) was significantly different between patients with a low cfDNA concentration (≤17.91 ng/ml) and those with a high cfDNA concentration (> 17.91 ng/ml). **d** Patients with a low cfDNA concentration had a higher 1-year overall survival rates (94% versus 56%, *P* < 0.0001) than those with a high cfDNA concentration. **e** An early decrease in cfDNA was related to a good therapeutic effect in two typical mCRC patients

The median concentration of cfDNA was 10.4 ng/ml (2.2–33.5 ng/ml), and the median content of cfDNA was 105 ng (8.8–438.4 ng). The results showed that the concentration of cfDNA was significantly correlated with the levels of CEA, CA199, CA125, NSE, LDH and the sum of tumor diameters, especially CEA (*P* < 0.0001, Pearson *r* = 0.81), LDH (*P* < 0.0001, Pearson *r* = 0.84) and the sum of tumor diameters (*P* < 0.0001, Pearson *r* = 0.80). The cfDNA content was strongly correlated with the levels of CEA (*P* < 0.0001, Pearson *r* = 0.84), LDH (*P* < 0.0001, Pearson *r* = 0.86) and the sum of tumor diameters (*P* < 0.0001, Pearson *r* = 0.76) (Table 4 and Fig. 2a). The mutational frequency in plasma was moderately correlated with the levels of CA199 (*P* < 0.0001, Pearson *r* = 0.66), LDH (*P* < 0.0001, Pearson *r* = 0.61) and the sum of tumor diameters (*P* < 0.0001, Pearson *r* = 0.64) (Table 4). The optimal cutoff level for cfDNA concentration was determined to be 17.91 ng/ml based on the 9-month PFS through ROC curve analysis (Fig. 2b). The patients with a low cfDNA concentration (≤17.91 ng/ml) had a longer PFS (11.7 months versus 6.6 months, *P* < 0.0001) and a higher 1-year overall survival rate (94% versus 56%, *P* < 0.0001) than those with a high cfDNA concentration (>17.91 ng/ml) (Fig. 2c and d). Two typical mCRC patients in this study were included, and the changes in cfDNA concentration were correlated with the effect of therapy (Fig. 2e).

**Table 4:** Correlation between cfDNA and clinical tumor burden

Tumor burden	cfDNA concentration (ng/ml)		cfDNA content (ng)		Mutational frequency (%)	
	***P*** value	Pearson r(95%CI)	***P*** value	Pearson r(95%CI)	***P*** value	Pearson r(95%CI)
CEA (ng/ml)	< 0.0001	0.81 (0.74–0.87)	< 0.0001	0.84 (0.78–0.89)	< 0.0001	0.56 (0.35–0.72)
CA199 (U/ml)	< 0.0001	0.66 (0.54–0.75)	< 0.0001	0.63 (0.50–0.73)	< 0.0001	0.66 (0.48–0.79)
CA125 (U/ml)	< 0.0001	0.59 (0.44–0.71)	< 0.0001	0.65 (0.51–0.75)	0.0036	0.42 (0.15–0.63)
NSE (ng/ml)	< 0.0001	0.65 (0.49–0.77)	< 0.0001	0.67 (0.51–0.78)	0.0022	0.50 (0.20–0.71)
LDH (U/L)	< 0.0001	0.84 (0.78–0.89)	< 0.0001	0.86 (0.80–0.90)	< 0.0001	0.61 (0.39–0.76)
Sum of diameter (mm)	< 0.0001	0.80 (0.72–0.85)	< 0.0001	0.76 (0.68–0.83)	< 0.0001	0.64 (0.46–0.77)

### Mutational status and tumor metastasis

**Fig. 3:**
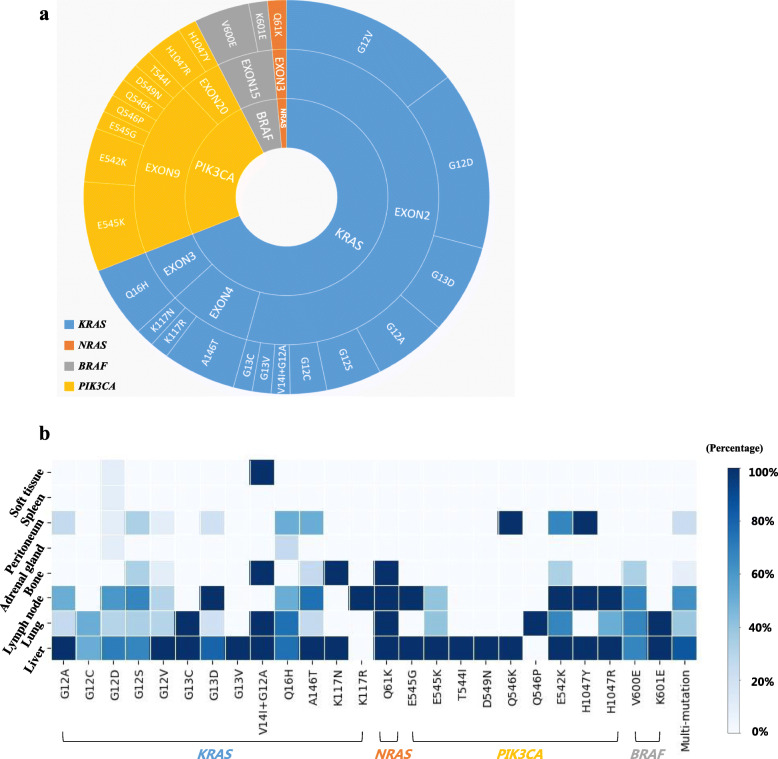
Mutational status and tumor metastasis. **a** Mutations detected in plasma. **b** The liver, lung and lymph nodes were the most common metastatic sites in mCRC regardless of the mutational status

The most common metastatic site was the liver (77.8%), followed by the lymph nodes (62.7%), lung (40.5%), peritoneum (14.3%) and bone (10.3%), in all patients. Other rare metastatic sites included the adrenal gland, the spleen and some soft tissues. Whether the gene status was wild-type or mutated, the sites of metastasis were similar (Supplemental Table 3). In the patients with gene mutations, the top three metastatic sites were also the liver, lymph nodes and lung, regardless of the type of mutation. Bone metastasis was often found in patients with G12 mutations in exon 2 of *KRAS*, A146 and K117 mutations in exon 4 of *KRAS*, Q61 mutations in exon 3 of *NRAS*, E542 mutations in exon 9 of *PIK3CA* and V600 mutations in exon 15 of *BRAF.* Peritoneal metastasis was mainly found in patients with *KRAS* or *PIK3CA* mutations (Fig. 3b).

## Discussion

In mCRC, patients harboring *RAS* and *BRAF* mutations do not benefit from anti-EGFR therapies, such as cetuximab [12–14]. Due to the heterogeneity of tissue samples, the mutational status may be different intralesionally and interlesionally. In recent years, analyzing mutations in plasma-derived ctDNA has been used to provide a more comprehensive overview of the mutational landscape compared to analyses of primary and/or metastatic tumors. Consequently, ctDNA analysis, as an alternative approach with a minimally invasive nature, has been gradually used to guide management and monitor treatment response in some cancers, including CRC [3, 15]. In this study, mutations in plasma were measured in 126 Chinese patients with mCRC by a panel of 4 genes (197 hotpots), including *KRAS, NRAS, BRAF,* and *PIK3CA*, using a NGS approach.

Our results showed that the concordance rate of *RAS/BRAF* mutations between plasma and tissue was 78.6%, which was in line with that found in previous studies [15–17]. In a subgroup of 92 patients from the CAPRI-GOIM clinical trial, NGS analysis of both tumor tissue and plasma identified a concordance rate of 78.3% [18], similar to our results. In our study, 11 patients had mutations in tissue that were not detected in plasma. Nine of these patients had a low tumor burden and their cfDNA concentrations ranged from 4 to 10 ng/ml, which was less than the median concentration. A low amount of tumor DNA could hypothetically lead to false-negative results in plasma. In the other two patients with high tumor load, their metastatic sites were predominantly the lymph nodes, bone and peritoneum. In such patients, these metastatic tumors might release less DNA into the circulation than metastatic tumors at other sites. For the subgroup of 16 patients with mutations in plasma but no mutations in tissue, we propose three possible reasons. First, the discrepancy may have resulted from the different sampling times and heterogeneity of tissue specimens. Eleven of these patients had their primary tumor removed before blood sample collection. Therefore, the testing results in plasma might have indicated the mutational status of metastases originating from a different clone. Indeed, the mutational signature of *KRAS* has been reported to be different between primary tumors and metastatic lesions, with an inconsistency rate ranging from 5 to 32% [19]. Second, sequencing analysis was performed by different approaches in our routine clinical work-up. Mutations in plasma were measured by a panel covering 197 hotpot mutations using NGS approach, while mutations in tissue were assayed by a panel containing only 17 hotpots using ARMS technology. Some rare mutations detected in plasma, such as *KRAS p.V14I, BRAF p.K601E*, and *PIK3CA p.E545G*, were not covered by the sequencing panel of tissue. Third, the sensitivity of variant detection used in plasma was 0.2%, while the ARMS technology used in tissue had an analytical sensitivity of approximately 1%, which could also have led to false-negative results in tissue.

How can anti-EGFR treatment be guided using the testing results from both plasma and tissue? It is generally believed that patients with any *RAS/BRAF* mutations in either tumor tissue or plasma have less treatment benefit than patients who have no mutations. A prospective multicenter clinical study [20] reported that 59, 11.8 and 14.4% of *KRAS*, *NRAS* and *BRAF* mutations were found in plasma, while 44, 8.8 and 7.2% were found in tissue respectively. Even patients who showed *RAS* wild-type in tissue failed to respond to anti-EGFR therapy when mutations were present in their plasma, with the incidence of disease progression as high as 56% [20]. Therefore, in our study, the majority of these patients with *RAS/BRAF* mutations in tissue and/or in plasma did not receive anti-EGFR treatment, except one (patient No. 25, in Supplemental Table 4). After surgical removal of this patient’s *RAS* wild-type primary tumor, only a small number of *KRAS*-mutated subclones were found in his plasma with a mutational frequency of 0.26%. Considering that there were only a few mutated clones in his plasma, we treated him with cetuximab plus chemotherapy, and the PFS was up to 12 months, indicating that he could still have clinical benefit from anti-EGFR therapy. Therefore, whether low frequency mutation in plasma (0.2–1%) is a contraindication for anti-EGFR treatment needs further study. In addition, polyclonal mutations were more frequently detected in plasma than in tissue (10.3% versus 0.9% in our study), which emerged mostly in the form of double mutations in *KRAS/PIK3CA* or *NRAS/PIK3CA*. Such polyclonal mutations may influence mechanisms of resistance, as reported in some other studies [21]. Consequently, the baseline assessment of the mutational status in plasma can identify additional mutated patients and further improve patient selection for anti-EGFR treatment.

This study further analyzed the correlation between cfDNA and tumor burden. We found that cfDNA concentration was positively correlated with the serological levels of CEA, CA199, NSE, LDH, and the sum of tumor diameters (including all tumors assessable). However, the correlation might not be tumor-specific because cfDNA comprises both healthy and tumor DNA. Therefore, we further found that the mutational frequency in plasma was also correlated with the levels of CA199 and LDH and the sum of tumor diameters. It was indicated that cfDNA concentration and mutational frequency could serve as quantitative tools for assessing tumor burden, which has been supported by some other studies [4]. In this study, we also found that patients with a higher cfDNA concentration had shorter PFS and OS than those with a lower cfDNA concentration, with an optimal threshold of 17.91 ng/ml. Another potential application of cfDNA testing is to dynamically evaluate therapeutic effects and predict recurrence [7]. As seen in Fig. 2e, we found that an early decrease in cfDNA concentration was related to a good therapeutic effect. Thomsen et al. also reported that a low level of cfDNA after chemotherapy, prior to radiological imaging evaluation, was associated with a low risk of progression [22].

In this study, the liver, lung and lymph nodes were the most common metastatic sites in mCRC regardless of specific mutations. Additionally, we also observed some interesting metastatic patterns in patients harboring rare mutations in this study. For example, one patient (patient No. 40 in Supplemental Table 4) with a high-frequency (28%) Q61K mutation in exon 3 of *NRAS* had mandible metastasis, which is a rare metastatic site in clinical practice. Patient No. 31, carrying both the *KRAS* p.V14I and *KRAS* p.G12A mutations, had metastasis mainly in soft tissues, such as the chest wall, anterior sacrum, and psoas major muscle. Patient No. 39, with the K117R mutation in exon 4 of *KRAS,* had only systemic lymph node metastases, and his cfDNA concentration was more than 50 ng/ml, but his PFS was more than 20 months. We believe that these patients with rare mutations might have unique mechanisms of metastasis that further influence survival, which might be an interesting problem worthy of further research.

There are some limitations of this study. First, correlations between rare mutations and metastatic sites could not be made because of the small sample size. Second, the mutational status in tissue and plasma were sequenced with panels used in our daily routine practice, covering only four genes, and these panels need to be optimized to obtain more information with low cost for every patient.

## Conclusion

In conclusion, our results suggest that analyzing mutations in plasma could provide a more comprehensive overview of the mutational landscape than analyzing mutations in tissue and that cfDNA concentration could be a quantitative biomarker to evaluate tumor burden and predict the survival of mCRC patients. The liver, lung and lymph nodes were the most common metastatic sites in mCRC regardless of the types of mutation.

## Supplementary information

**Additional file 1: Supplemental Table 1**. The testing panel covering a total of 197 hotspots in plasma. **Supplemental Table 2**. The testing panel covering a total of 17 hotspots in tissue. **Supplemental Table 3**. Mutational status in plasma and metastatic sites. **Supplemental Table 4**. Mutational status and metastatic sites of all the 126 patients.

## Data Availability

The datasets used and/or analyzed during the current study are available from the corresponding author on reasonable request. Some important information about the mutational status and metastatic sites of all the 126 patients has been presented in the Supplemental Table 4.

## Abbreviations

ctDNA: Circulating tumor DNA
cfDNA: Circulating free DNA
mCRC: Metastatic colorectal cancer
NGS: Next-generation sequencing
ARMS: Amplification-refractory mutation system
CEA: Carcinoembryonic antigen
CA199: Carbohydrate antigen 199
CA125: Carbohydrate antigen 125
NSE: Neuron-specific enolase
LDH: Lactate dehydrogenase
PET/CT: Positron emission tomography/computed tomography
PFS: Progression-free survival
OS: Overall survival
EGFR: Epidermal growth factor receptor
RECIST: Response evaluation criteria in solid tumors
FFPE: Formalin-fixed paraffin-embedded
ROC: Receiver operating curve
