# Supplementary material for: Role of circulating free DNA in evaluating clinical tumor burden and predicting survival in Chinese metastatic colorectal cancer patients
Source: BMC Cancer. 2020 Oct 16;20:1006. doi: 10.1186/s12885-020-07516-7 (PMC7566057; doi:10.1186/s12885-020-07516-7)
Supplement: Supplementary file 1 — Additional file 1: Supplemental Table 1. The testing panel covering a total of 197 hotspots in plasma. Supplemental Table 2. The testing panel covering a total of 17 hotspots in tissue. Supplemental Table 3. Mutational status in plasma and metastatic sites. Supplemental Table 4. Mutational status and metastatic sites of all the 126 patients. [file 12885_2020_7516_MOESM1_ESM.docx]

**Supplemental Table 1: The testing panel covering a total of 197 hotspots in plasma**

| *BRAF* exon15 | G606S | D594N | D594V | L597R | V600L | G596R | N581S |
| --- | --- | --- | --- | --- | --- | --- | --- |
|  | V600E | G606R | T589I | D594G | L597Q | D594H | V600Efs*11 |
|  | V600M | V600G | K601N | D587E | D594A | N58I | V600_k601delinsE |
|  | F595L | T599I | V600Q | K601E | D587A | N581T |  |
| *KRAS* exon2,3,4 | Q150* | G13D | Q61L | A146V | G13R | A59E | A134V |
|  | E143K | G13A | G60V | S136N | G13C | E49* | E98* |
|  | A134T | G12E | T58I | K117R | G12D | E31K | T58_M72dup |
|  | R97I | G12R | P34L | Y71C | G12L | Q22* | E62_A66dup |
|  | R68S | G12C | L23I | E63K | G10E | V14I | C51_S65dup |
|  | Q61R | G10R | L19F | Q61P | A146P | G13V | A59S |
|  | Q61K | R149G | G13E | A59G | R135K | G13K | E49K |
|  | A59T | G138E | G13F | D57N | N116H | G13S | G13_V14delinsDI |
|  | T35I | K117N | G12V | D33E | Q70P | G12A | A11_G12dup |
|  | L23R | D92Y | G12F | Q22R | Q61H | G12I | L19_T20delinsFA |
|  | T20M | Y64H | G12S | V14G | Q61E | A146T | K16_S17insW |
|  | G12W | G12Y | Q22K | D30E |  |  |  |
| *NRAS* exon2,3,4 | A91V | R68S | A66T | Q61H | Q61L | A146V | G12S |
|  | Q61R | Q61E | Q61K | T20I | G13V | A146P | V9A |
|  | G13D | G13C | G13R | G12V | G12A | A146T | K117N |
|  | G12D | G12C | G12R |  |  |  |  |
| *PIK3CA* exon9,20 | E522K | A1046T | S1015Y | E545A | G1049A | N1044K | E547K |
|  | L540I | H1047L | T1025N | E545D | T536A | H1047P | G1007R |
|  | E542G | G1049R | M1043I | Q546L | E542Q | H1048Q | R10223Q |
|  | E545K | E522G | A1046E | W552* | T544N | W1051* | M1043V |
|  | E545G | S541T | H1047Q | A1020V | Q546K | H1065Y | D1045N |
|  | 546P | E542V | G1049D | A1035V | Q546H | P539R | H1047R |
|  | D549N | E545Q | R524K | N1044D | W552C | E542A | G1049S |
|  | S1008P | E545V | E542K | H1047Y | Y1021C | T544I | T1052K |
|  | T1025A | Q546R | I543N | H1048Y | Y1038C | Q546E | N1068Kfs*5 |
|  | M1043T | L551P |  |  |  |  |  |

**Supplemental Table 2: The testing panel covering a total of 17 hotspots in tissue**

| **Gene** | **Exon** | **Mutation** | **Nucleotide changes** | **Cosmic ID** |
| --- | --- | --- | --- | --- |
| *KRAS* | 2 | p.G12S | c.34G>A | COSM517 |
|  |  | p.G12D | c.35G>A | COSM521 |
|  |  | p.G12C | c.34G>T | COSM516 |
|  |  | p.G12V | c.35G>T | COSM520 |
|  |  | p.G12A | c.35G>C | COSM522 |
|  |  | p.G13D | c.38G>A | COSM532 |
|  | 3 | p.Q61H | c.183A>C | COSM554 |
|  | 4 | p.K117R | c.351A>C | COSM19940 |
|  |  | p.K117N | c.351A>T | COSM28519 |
|  |  | p.A146T | c.436G>A | COSM19404 |
|  |  | p.A146V | c.437C>T | COSM19900 |
|  |  | p.A146P | c.436G>C | COSM19905 |
| *NRAS* | 2 | p.G12D | c.35G>A | COSM564 |
|  | 3 | p.Q61R | c.182A>G | COSM584 |
|  |  | p.Q61K | c.181C>A | COSM580 |
| *PIK3CA* | 20 | p.H1047R | c.3140A>G | COSM775 |
| *BRAF* | 15 | p.V600E | c.1799T>A | COSM476 |

**Supplemental Table 3: Mutational status in plasma and metastatic sites**

| Metastatic sites | No. (%) | | *P* value |
| --- | --- | --- | --- |
|  | Wild-type (N=69) | Mutated (N=57) |  |
| Liver | 51（73.9） | 47（82.5） | 0.25 |
| Lymph node | 38（55.1） | 41（71.9） | 0.05 |
| Lung | 30（43.5） | 21（36.8） | 0.45 |
| Peritoneum | 8（11.6） | 10（17.5） | 0.34 |
| Bone | 6（8.7） | 7（12.3） | 0.51 |
| Soft tissue | 4（5.8） | 2（3.5） | 0.55 |
| Adrenal gland | 2（2.9） | 2（3.5） | 0.85 |
| Bladder | 2（2.9） | 0（0） | 0.19 |
| Spleen | 0（0） | 1（1.8） | 0.27 |

**Supplemental Table 4: Mutational status and metastatic sites of all the 126 patients**

| No. | Primary site | Mutation in tissue | Mutation in plasma | Mutational hotspot | Metastatic sites |
| --- | --- | --- | --- | --- | --- |
| 1 | Descending colon | BRAF EXON15 | BRAF EXON15 | V600E | Liver; Lymph node |
| 2 | Sigmoid colon | BRAF EXON15 | BRAF EXON15 | V600E | Liver; Lung; Lymph node |
| 3 | Descending colon | / | BRAF EXON15 | K601E | Liver; Lymph node |
| 4 | Sigmoid colon | / | BRAF EXON15 | V600E | Lung; Bone |
| 5 | Sigmoid colon | KRAS EXON2 | KRAS EXON2 | G12S | Liver; Lung; Lymph node |
| 6 | Sigmoid colon | KRAS EXON2 | KRAS EXON2 | G12V | Liver; Lymph node |
| 7 | Sigmoid colon | KRAS EXON2 | KRAS EXON2 | G12A | Liver; Lymph node |
| 8 | Rectum | KRAS EXON2 | KRAS EXON2 | G12D | Liver; Lymph node |
| 9 | Rectum | KRAS EXON2 | KRAS EXON2 | G12V | Liver; Lymph node |
| 10 | Rectum | KRAS EXON2 | KRAS EXON2 | G12D | Liver; Lymph node |
| 11 | Rectum | KRAS EXON2 | KRAS EXON2 | G12D | Liver; Lymph node |
| 12 | Rectum | KRAS EXON2 | KRAS EXON2 | G13D | Lymph node; Peritoneum |
| 13 | Rectum | KRAS EXON2 | KRAS EXON2 | G12C | Lung; Lymph node |
| 14 | Rectum | KRAS EXON2 | KRAS EXON2 | G12S | Liver; Bone |
| 15 | Rectum | KRAS EXON2 | KRAS EXON2 | G13D | Liver; Lymph node |
| 16 | Rectum | KRAS EXON2 | KRAS EXON2 | G12V | Liver; Lymph node |
| 17 | Ascending colon | KRAS EXON2 | KRAS EXON2 | G12A;G12D | Liver; Lung |
| 18 | Ascending colon | KRAS EXON2 | KRAS EXON2 | G12D | Lymph node; Adrenal gland; Spleen |
| 19 | Transverse colon | KRAS EXON2 | KRAS EXON2 | G12V | Liver |
| 20 | Transverse colon | KRAS EXON2 | KRAS EXON2 | G12D | Liver; Lung; Lymph node; Peritoneum |
| 21 | Descending colon | KRAS EXON2 | KRAS EXON2 | G12C | Liver |
| 22 | Sigmoid colon | KRAS EXON2 | KRAS EXON2 | G13D | Liver; Lung; Lymph node |
| 23 | Rectum | KRAS EXON2 | KRAS EXON2 | G12D | Liver; Lung; Lymph node |
| 24 | Ascending colon | / | KRAS EXON2 | G12D | Liver; Lymph node |
| 25 | Transverse colon | / | KRAS EXON2 | G13C | Liver; Lung |
| 26 | Sigmoid colon | / | KRAS EXON2 | G12V | Liver; Lung |
| 27 | Rectum | / | KRAS EXON2 | G12D | Lung |
| 28 | Rectum | / | KRAS EXON2 | G13V | Liver |
| 29 | Ascending colon | / | KRAS EXON2 | G12V | Liver; Lung; Bone; Peritoneum |
| 30 | Ascending colon | / | KRAS EXON2 | G12S | Lymph node; Peritoneum |
| 31 | Rectum | / | KRAS EXON2 | V14I;G12A | Liver; Lung; Bone; Soft tissue |
| 32 | Rectum | / | KRAS EXON2 | G12D | Lymph node; Soft tissue |
| 33 | Sigmoid colon | KRAS EXON4 | KRAS EXON3 | Q61H | Liver; Lung; Lymph node; Adrenal gland; Peritoneum |
| 34 | Sigmoid colon | / | KRAS EXON3 | Q61H | Liver |
| 35 | Rectum | KRAS EXON4 | KRAS EXON4 | A146T | Liver; Lymph node |
| 36 | Sigmoid colon | KRAS EXON4 | KRAS EXON4 | A146T | Liver; Lymph node |
| 37 | Rectum | KRAS EXON4 | KRAS EXON4 | A146T | Liver; Lymph node; Peritoneum |
| 38 | Transverse colon | / | KRAS EXON4 | K117N | Liver; Lymph node; Bone |
| 39 | Transverse colon | / | KRAS EXON4 | K117R | Lymph node |
| 40 | Transverse colon | NRAS EXON3 | NRAS EXON3 | Q61K | Liver; Lung; Lymph node; Bone |
| 41 | Sigmoid colon | / | PIK3CA EXON20 | H1047R | Liver; Lung; Lymph node |
| 42 | Sigmoid colon | / | PIK3CA EXON9 | E545G | Liver; Lymph node |
| 43 | Ascending colon | PIK3CA EXON9 | PIK3CA EXON9 | Q546K | Liver; Peritoneum |
| 44 | Sigmoid colon | PIK3CA EXON9 | PIK3CA EXON9 | D549N | Liver; Lymph node |
| 45 | Sigmoid colon | PIK3CA EXON9 | KRAS EXON4 | A146T | Liver; Lung; Lymph node; Bone; Peritoneum |
|  |  |  | PIK3CA EXON9 | E542K |  |
| 46 | Rectum | KRAS EXON3 | KRAS EXON3 | Q61H | Liver; Lung; Lymph node; Peritoneum |
|  |  |  | PIK3CA EXON9 | E542K |  |
| 47 | Ascending colon | KRAS EXON2 | KRAS EXON2 | G13D | Liver; Lymph node |
|  |  | PIK3CA EXON20 | PIK3CA EXON20 | H1047R |  |
| 48 | Ascending colon | KRAS EXON2 | KRAS EXON2 | G12V | Liver; Lung |
|  |  |  | PIK3CA EXON9 | E545K |  |
| 49 | Ascending colon | KRAS EXON2 | KRAS EXON2 | G12A | Liver; Lymph node; Peritoneum |
|  |  |  | PIK3CA EXON20 | H1047Y |  |
| 50 | Ascending colon | KRAS EXON2 | KRAS EXON2 | G13D | Liver; Lymph node |
|  |  |  | PIK3CA EXON20 | E545 |  |
| 51 | Descending colon | KRAS EXON2 | KRAS EXON2 | G12V | Liver; Lung |
|  |  |  | PIK3CA EXON9 | E545K |  |
| 52 | Sigmoid colon | KRAS EXON2 | KRAS EXON2 | G12A | Liver |
|  |  |  | PIK3CA EXON9 | E545K |  |
| 53 | Rectum | KRAS EXON2 | KRAS EXON2 | G12V | Liver; Lymph node |
|  |  |  | PIK3CA EXON9 | E542K |  |
| 54 | Rectum | KRAS EXON2 | KRAS EXON3 | Q61H | Lung; Lymph node |
|  |  |  | PIK3CA EXON9 | Q546P |  |
| 55 | Rectum | KRAS EXON2 | KRAS EXON2 | G12D | Liver; Lymph node |
|  |  |  | PIK3CA EXON9 | E545K |  |
| 56 | Ascending colon | KRAS EXON2 | KRAS EXON2 | G12V | Liver |
|  |  |  | PIK3CA EXON9 | T544I |  |
| 57 | Ascending colon | NRAS EXON2 | NRAS EXON2 | G13D | Lymph node |
|  |  |  | KRAS EXON3 | A59T |  |
|  |  |  | PIK3CA EXON20 | A1035V |  |
| 58 | Sigmoid colon | BRAF EXON15 | / | / | Lymph node; Peritoneum |
| 59 | Sigmoid colon | KRAS EXON2 | / | / | Liver; Lymph node |
| 60 | Sigmoid colon | KRAS EXON2 | / | / | Liver; Lung |
| 61 | Rectum | KRAS EXON2 | / | / | Lung |
| 62 | Rectum | KRAS EXON2 | / | / | Liver; Lung; Lymph node |
| 63 | Ascending colon | KRAS EXON2 | / | / | Liver; Lung |
| 64 | Descending colon | KRAS EXON2 | / | / | Liver |
| 65 | Rectum | KRAS EXON2 | / | / | Lung; Lymph node |
| 66 | Rectum | NRAS EXON2 | / | / | Liver; Lymph node |
| 67 | Sigmoid colon | KRAS EXON3 | / | / | Liver; Lymph node |
| 68 | Sigmoid colon | PIK3CA EXON20 | / | / | Liver; Lymph node; Bone; Bladder |
| 69 | Ascending colon | / | / | / | Lung |
| 70 | Ascending colon | / | / | / | Liver; Lung; Lymph node; Peritoneum |
| 71 | Transverse colon | / | / | / | Liver; Adrenal gland; Peritoneum |
| 72 | Descending colon | / | / | / | Lung; Bone; Adrenal gland |
| 73 | Descending colon | / | / | / | Liver; Lymph node |
| 74 | Sigmoid colon | / | / | / | Liver |
| 75 | Sigmoid colon | / | / | / | Liver |
| 76 | Sigmoid colon | / | / | / | Liver; Lymph node |
| 77 | Sigmoid colon | / | / | / | Liver; Lymph node |
| 78 | Sigmoid colon | / | / | / | Lung; Lymph node |
| 79 | Sigmoid colon | / | / | / | Liver; Lymph node |
| 80 | Sigmoid colon | / | / | / | Liver; Lung; Lymph node |
| 81 | Sigmoid colon | / | / | / | Liver |
| 82 | Sigmoid colon | / | / | / | Liver; Lymph node |
| 83 | Sigmoid colon | / | / | / | Lung; Lymph node; Bone; Soft tissue |
| 84 | Sigmoid colon | / | / | / | Liver; Lymph node; Soft tissue |
| 85 | Sigmoid colon | / | / | / | Liver |
| 86 | Sigmoid colon | / | / | / | Liver; Lung; Lymph node |
| 87 | Sigmoid colon | / | / | / | Liver; Lung; Lymph node |
| 88 | Sigmoid colon | / | / | / | Liver |
| 89 | Sigmoid colon | / | / | / | Liver |
| 90 | Sigmoid colon | / | / | / | Liver; Lung; Lymph node |
| 91 | Sigmoid colon | / | / | / | Liver; Lymph node; Peritoneum |
| 92 | Sigmoid colon | / | / | / | Lung; Lymph node; Bone; Peritoneum |
| 93 | Sigmoid colon | / | / | / | Liver; Lymph node; Bone |
| 94 | Sigmoid colon | / | / | / | Liver |
| 95 | Sigmoid colon | / | / | / | Lung; Lymph node |
| 96 | Sigmoid colon | / | / | / | Lung; Lymph node |
| 97 | Sigmoid colon | / | / | / | Liver; Lymph node |
| 98 | Sigmoid colon | / | / | / | Liver |
| 99 | Sigmoid colon | / | / | / | Liver |
| 100 | Sigmoid colon | / | / | / | Lung; Lung; Peritoneum |
| 101 | Sigmoid colon | / | / | / | Liver; Lymph node |
| 102 | Sigmoid colon | / | / | / | Lung; Lymph node; Peritoneum |
| 103 | Rectum | / | / | / | Liver |
| 104 | Rectum | / | / | / | Lung; Lymph node |
| 105 | Rectum | / | / | / | Liver; Lymph node |
| 106 | Rectum | / | / | / | Lung; Lymph node |
| 107 | Rectum | / | / | / | Liver |
| 108 | Rectum | / | / | / | Lung |
| 109 | Rectum | / | / | / | Liver; Lymph node |
| 110 | Rectum | / | / | / | Lung |
| 111 | Rectum | / | / | / | Live; Lymph node |
| 112 | Rectum | / | / | / | Bladder |
| 113 | Rectum | / | / | / | Liver; Lung |
| 114 | Rectum | / | / | / | Liver |
| 115 | Rectum | / | / | / | Liver |
| 116 | Rectum | / | / | / | Liver; Lung; Lymph node |
| 117 | Rectum | / | / | / | Liver; Lung; Lymph node; Bone |
| 118 | Rectum | / | / | / | Liver; Peritoneum |
| 119 | Rectum | / | / | / | Liver |
| 120 | Rectum | / | / | / | Lung; soft tissue |
| 121 | Rectum | / | / | / | Liver; Lymph node; soft tissue |
| 122 | Rectum | / | / | / | Liver; Lung; Lymph node |
| 123 | Rectum | / | / | / | Lung |
| 124 | Ascending colon | / | / | / | Liver |
| 125 | Ascending colon | / | / | / | Liver |
| 126 | Ascending colon | / | / | / | Liver; Lung |
